# Urban air pollution and climate change: “The Decalogue: Allergy Safe Tree” for allergic and respiratory diseases care

**DOI:** 10.1186/s12948-018-0098-3

**Published:** 2018-09-11

**Authors:** Vincenzo Patella, Giovanni Florio, Diomira Magliacane, Ada Giuliano, Maria Angiola Crivellaro, Daniela Di Bartolomeo, Arturo Genovese, Mario Palmieri, Amedeo Postiglione, Erminia Ridolo, Cristina Scaletti, Maria Teresa Ventura, Anna Zollo, Air Pollution and Climate Change Task Force of the Italian Society of Allergology, Asthma and Clinical Immunology (SIAAIC)

**Affiliations:** 1 Division Allergy and Clinical Immunology, Department of Medicine ASL Salerno, “Santa Maria della Speranza” Hospital, Battipaglia, Salerno, Italy; 2 Postgraduate Program in Allergy and Clinical Immunology–University of Naples Federico II, Naples, Italy; 3 Air Pollution and Climate Change Task Force of the Italian Society of Allergology, Asthma and Clinical Immunology (SIAAIC), Milan, Italy; 4 Laboratory of Environmental Analysis, Department of Public Health, ASL Salerno, Salerno, Italy; 5 Department of Cardiac, Thoracic and Vascular Sciences, University of Padua, Padua, Italy; 6 Association of International Culture, Athena of Paestum, Capaccio-Paestum, Salerno, Italy; 7 Former Primary of Unit of Pediatry, Hospital of Eboli, Salerno, Italy; 8 International Court of the Environment Foundation (ICEF), Rome, Italy; 9 Department of Medicine and Surgery, University of Parma, Parma, Italy; 10 Unit of Internal Medicine, Department of Experimental and Clinical Medicine, University of Florence, Florence, Italy; 11 Department of Interdisciplinary Medicine, University of Bari, Bari, Italy; 12 Department of Studies and Researches, Movimento Ecologista Europeo FareAmbiente, Rome, Italy

## Abstract

**Background:**

According to the World Health Organization, air pollution is closely associated with climate change and, in particular, with global warming. In addition to melting of ice and snow, rising sea level, and flooding of coastal areas, global warming is leading to a tropicalization of temperate marine ecosystems. Moreover, the effects of air pollution on airway and lung diseases are well documented as reported by the World Allergy Organization.

**Methods:**

Scientific literature was searched for studies investigating the effect of the interaction between air pollution and climate change on allergic and respiratory diseases.

**Results:**

Since 1990s, a multitude of articles and reviews have been published on this topic, with many studies confirming that the warming of our planet is caused by the “greenhouse effect” as a result of increased emission of “greenhouse” gases. Air pollution is also closely linked to global warming: the emission of hydrocarbon combustion products leads to increased concentrations of biological allergens such as pollens, generating a mixture of these particles called particulate matter (PM). The concept is that global warming is linked to the emission of hydrocarbon combustion products, since both carbon dioxide and heat increase pollen emission into the atmosphere, and all these particles make up PM10. However, the understanding of the mechanisms by which PM affects human health is still limited. Therefore, several studies are trying to determine the causes of global warming. There is also evidence that increased concentrations of air pollutants and pollens can activate inflammatory mediators in the airways. Our Task Force has prepared a Decalogue of rules addressing public administrators, which aims to limit the amount of allergenic pollen in the air without sacrificing public green areas.

**Conclusions:**

Several studies underscore the significant risks of global warming on human health due to increasing levels of air pollution. The impact of climate change on respiratory diseases appears well documented. The last decades have seen a rise in the concentrations of pollens and pollutants in the air. This rise parallels the increase in the number of people presenting with allergic symptoms (e.g., allergic rhinitis, conjunctivitis, and asthma), who often require emergency medical care. Our hope is that scientists from different disciplines will work together with institutions, pharmaceutical companies and lay organizations to limit the adverse health effects of air pollution and global warming.

## Background

Climate change is already underway and is expected to continue in the next decades: temperatures are increasing, global precipitation patterns are changing, ice and snow are melting, and global average sea level is rising. Moreover, the impact of global warming on human health has been documented by several reports from the World Health Organization (WHO) [1], which illustrate that a major concern about this phenomenon is the increase in air pollution levels. In this respect, the last report of the European Environment Agency (EEA) indicates that up to 96% of the urban population in the European Union are exposed to particulate and fine dust levels in excess of threshold values [2]. In addition to air pollution produced by hydrocarbon combustion emissions, there is another issue: the formation of particles related to global warming, and the presence of gases, such as ozone, with generation of a mixture of particles in the atmosphere, called particulate matter (PM) or particle pollution. Environmental PM is a heterogeneous mixture of particles produced by various processes, whose size ranges from 2.5 to 10 μm. The fine particulate fraction (PM2.5) (also known as “fine particles”) consists of particles with a size of 2.5 microns (or smaller), while the ultrafine particulate fraction (also known as “ultrafine particles”) consists of particles with a size smaller than 0.1 μm. The latter has a variable chemical composition, with the highest volatility, which is obviously due to its smaller size. It should also be noted that, although the ultrafine fraction represents less than 1% of the particulate mass, it is suspected of causing the greatest harm to the exposed population [3–6]. Importantly, this particulate fraction can reach the deepest parts of the bronchial tree, leading to major inflammatory and procarcinogenic effects on the bronchial mucosa [6]. Of note, PM is also involved in other diseases, including cardiovascular disorders such as coronary heart disease and heart failure. These conditions are mainly due to the oxidative stress generated by the inflammatory response, with both short- and long-term adverse effects on human health through a variety of mechanisms [7–11]. In addition, it has been demonstrated that exposure to ozone and PM2.5 induces adverse health effects, including various pulmonary diseases and lung cancer. Overall, air pollution-related global mortality has been associated with several emission sources; therefore, air pollution control can be an effective strategy to reduce mortality [12]. Both concentrations and chemical composition of PM show a large variability not only geographic, but also seasonal. PM concentrations are usually higher in urban areas than in rural areas. With regard to seasonal variability, PM levels increase significantly during the winter due to increased emissions from road traffic and residential heating, and decrease during spring and summer when pollen concentrations are typically highest [13].

Some particulates are naturally occurring and originate from volcanoes, dust storms, forest and grassland fires, living vegetation, and sea spray. Although most aerosols remain suspended in the atmosphere for short periods (typically between 4 days and a week), they can travel vast distances. Dust plumes from the Sahara often cross the Atlantic and reach the Caribbean. Winds sweep a mixture of Asian aerosols, including dust from the Gobi desert and pollution from China, to the east over Japan and toward the central Pacific Ocean. Smoke from wildfires in Siberia and Canada can go as far as the Arctic ice cap [11]. Sea spray from the Mediterranean Sea and mineral dust from Sahara in North Africa can contribute to the chemical composition of PM in Southern Europe [14, 15].

Human activities, including the combustion of fossil fuels in vehicles, power plants, and many industrial processes [10], can also generate significant amounts of particulates. Coal combustion in developing countries is the primary means to generate energy and heat homes. Although salt spray is the most common form of particulate in the atmosphere over the oceans, anthropogenic aerosols, i.e. those that are generated by human activities, currently account for about 10% of the total mass of aerosols in the atmosphere [11].

In recent years, air quality has improved in industrialized Western countries, while it has worsened in developing countries. In Asia, for example, anthropogenic emissions have increased in recent decades with the rapid development of urbanization and industrialization. On the contrary, aerosol emissions have declined in North America and Europe with the relocation of factories to developing countries and with the adoption of more stringent clean air regulations in Western countries. However, it may be difficult to define the amount of air pollution in different areas of the world, since most aerosols remain suspended in the atmosphere for short periods, typically between 4 days and a week. PM particles in the atmosphere can move at 5 m (16.4 ft) per second, and may travel thousands of kilometres in a week, making it difficult to accurately measure pollution levels; consequently, any predictions about the deterioration of air quality can be distorted by this initial bias, especially with regard to time.

### Air pollution and climate change-related health impacts

Global warming is a major public health emergency. Global temperature has risen markedly over the last 50 years and is still rising, as demonstrated by warming of the oceans, rising sea levels, melting of glaciers, retreating of Arctic sea ice, and diminishing snow cover in the Northern Hemisphere. Climate change is also associated with extreme weather events such as heat waves, droughts, floods, and hurricanes. These changes are probably due to increased emissions of greenhouse gases (namely, carbon dioxide, methane and nitrous oxide), largely from anthropogenic sources. Projections of future greenhouse gas emissions indicate a worsening of the situation with further increase in average temperatures and temperature extremes by the end of the twenty-first century [16].

A large body of evidence suggests that climate change have significant health impacts, both direct and indirect. Direct health impacts include morbidity and mortality associated with direct exposure to hazardous weather conditions such as heat waves, floods, cyclones, etc. Indirect health impacts, which include the majority of climate change-related diseases, are mediated by environmental conditions which affect the spatial and temporal distribution of aeroallergens, the generation and dispersion of air pollutants, and the population of disease-transmitting vectors like mosquitoes and ticks.

Several epidemiological studies have demonstrated a close association between global warming, air pollution and respiratory allergic diseases, such as bronchial asthma and rhinitis. Climate changes affect the quantity, quality, timing and distribution of aeroallergens [17]. The increased atmospheric carbon dioxide concentrations due to fossil fuel burning stimulate the growth of plants, including allergenic species. On top of that, higher temperatures can lead to earlier and longer pollination seasons, leading to increased pollen sensitization rate and symptom duration. Individuals with asthma are at increased risk of developing obstructive airway attacks when exposed to gaseous and particulate components of air pollution [17]. In addition, air pollution can modify the allergenic potential of pollens, especially in the presence of favourable weather conditions. Air pollution—in particular PM, diesel exhaust particles (DEP), ozone, nitrogen dioxide, and sulfur dioxide—have been shown to have an inflammatory effect on the airways of susceptible subjects, causing increased mucosal permeability, facilitating the penetration and access of inhaled allergens to the cells of the immune system [18]. There are also data suggesting that air pollution can lead to the development of asthma. Therefore, climate change combined with air pollution may have severe impacts on respiratory health, especially in children.

Thunderstorms occurring during the pollen season have been shown to induce severe asthma attacks in pollinosis patients [18, 19]. The thunderstorm-related epidemics are limited to late spring and summer when there are high levels of pollen grains in the atmosphere. The main hypotheses for thunderstorm-related asthma claim that thunderstorms can concentrate pollen grains at ground level which rupture due to osmotic shock and release allergenic particles of respirable size into the atmosphere [19].

Rising temperatures, a higher concentration of carbon dioxide in the atmosphere, heavier rainfalls, and higher humidity induce faster proliferation of pollens, moulds, and fungi [20]. In addition, increases in humidity associated with higher temperatures may encourage the growth of cockroaches, house dust mites, and moulds, and, thus, allergen load [17]. Higher temperatures can also induce the migration of stinging and biting insects into new environments, as well as increasing the population of existing insect species [19]. Indeed, climate changes and globalization are altering the distribution and consistency of allergenic species including venomous insects. In particular, given the expansion of native species and the colonization of invasive ones, one can predict, despite the uncertainty of current models, that stinging Hymenoptera will pose new challenges in the future [21].

Many studies have reported the impact of climatic changes on the spatial and temporal distribution of infectious diseases such as vector-borne, rodent-borne, food-borne and water-borne diseases, including in particular malaria, tick-borne diseases, cholera and other diarrhoeal diseases. Importantly, several vector-borne diseases have emerged in Europe in recent years; these include vivax malaria, West Nile fever, dengue fever, Chikungunya fever, Leishmaniasis, Lyme disease, and tick-borne encephalitis [22, 23].

Chronic exposure to air pollution is also associated with pulmonary inflammation, chronic obstructive pulmonary disease (COPD), and lung cancer. Several studies suggest that chronic exposure to outdoor air pollution increases the prevalence and incidence of COPD [24]. Moreover, among individuals with COPD, air pollution is associated with increased respiratory symptoms, loss of lung function, COPD exacerbations and higher mortality [24]. Air pollution is also linked to lung cancer, and the International Agency for Research on Cancer (IARC) has classified the particulate matter in outdoor air pollution as carcinogenic to humans [25].

Studies from across the world have consistently demonstrated that exposure to particulate air pollution is associated with a host of cardiovascular diseases, including myocardial ischaemia and infarctions, heart failure, arrhythmias, strokes and increased cardiovascular mortality [26]. Acute exposure may contribute to plaque instability and trigger acute cardiovascular events, including myocardial ischaemia and infarction, stroke, heart failure, arrhythmias, and sudden death, particularly in high-risk subjects, while chronic exposure may favour the development and progression of atherosclerosis and possibly of hypertension in the long term [27].

Finally, a number of studies have reported an association between air pollution and higher risk of neuroinflammation and neurodegeneration [28, 29]. In particular, there is some evidence that chronic exposure to airborne pollutants in the early years of life may contribute to neurodegenerative disease processes including Alzheimer’s disease and Parkinson’s disease [30].

In summary, air pollution and climate change-related health impacts are enormous and are expected to increase during the next decades. As a result, also the social and economic costs of climate change are enormous and will increase in the future. Nevertheless, there is a relative scarcity of economic studies on the health impacts of climate change. Most studies have estimated the economic costs of extreme weather events or the impacts of climate change on respiratory diseases and infectious diseases (vector borne, water related, and food borne). However, these economic studies are heterogeneous in terms of objectives and methodology. To this end, a review has pointed out the need for climate change-specific health economic guidelines to provide a standardized approach and stimulate economic research [31]. A more recent review, conducted to evaluate the health costs resulting from human exposure to weather-related extreme events such as heat waves, droughts, floods, storms and hurricanes, has highlighted a lack of evidence about the potential cost-effectiveness of interventions that exploits the capacity of natural ecosystems to reduce exposure to extreme events, despite the observations that evidence that poor land management increases the vulnerability to these events [32]. However, all studies identified in this review indicate that the health costs associated with the impacts of extreme events are substantial. For example, six climate change-related events that struck the United States between 2000 and 2009, accounted for about $14 billion in lost lives and health costs [33].

### Mechanisms responsible for allergic and respiratory diseases

The mechanisms by which PM affects human health are still debated [34]. The atmosphere is the medium of transit not only for pollutant gases and particles, but also for a wide variety of biogenic materials of plant and animal origin. Among biogenic materials, there is the bioaerosol, which consists of different kinds of airborne particles such as viruses, bacteria, moulds, plant fibres, and also pollen with variable sizes, ranging from tens of nanometers to a few hundreds of micrometers. These biological substances are involved in allergic and respiratory diseases, and act both directly and indirectly on airway epithelial cells. The increased concentration of pollen and air pollutants can activate different epithelial and immune cells in the airways, leading to the release of inflammatory mediators [35]. However, the specific components and the mechanisms responsible for oxidative stress following exposure to PM are not fully understood. Direct oxidant production by air pollution particles in epithelial cells of the respiratory mucosa has been reported. This effect has been attributed to both organic components and a number of heavy metals (e.g., arsenic, cadmium, chromium, mercury, nickel, lead, and thallium). These metals have a wide distribution in the environment as a result of their multiple industrial, domestic, agricultural, medical, and technological applications, which raises many concerns about their potential effects on human health and the environment. The main consequences of oxidative stress include the activation of specific cellular reactions (including activation of kinases and transcription factors), the release of inflammatory mediators, and eventually cell damage or apoptosis [36]. All this promotes bronchial hyperreactivity as well as a decline in pulmonary function, increasing the risk of allergic sensitisation, asthma, chronic obstructive pulmonary disease, and cancer. Recent epigenetic studies have shown that a number of genes controlling immune and inflammatory responses are sensitive to tropospheric ozone. Indeed, atmospheric pollutants like diesel exhaust particles [particulate] (DEPs) and ozone are directly involved in asthma pathogenesis [37]. Importantly, DEPs are a major component of traffic-related particulate air pollution in most urban areas. In addition, a number of evidence indicates that DEPs may facilitate entry of allergens into the airways eliciting immunopathological response, and can also bind pollen, dog, cat, and house dust mite allergens [38–40]. Due to their very fine size, DEPs can facilitate the penetration of these allergens into the lungs, thereby increasing the amount of allergens interacting with the airway epithelium. It has also been demonstrated that DEPs can disrupt pollen particles causing the release of allergenic sub-pollen particles. Interestingly, a detailed analysis of pollen taken from air-polluted areas showed an increased amount of allergenic proteins with higher allergenic potential. In another study, comparing recent and decade-old pollen extracts, the recent pollen extract showed higher allergenic potency. In addition, the recent pollen extract obtained from the urban area had a higher allergenic potency than the recent pollen extract from the suburban area [39]. More recently, the same authors of the above study demonstrated that a recent pollen extract from an urban area significantly exposed to pollution and climate change was more effective at inducing transepithelial permeability and ROS production in cultured airway epithelial cells [41]. This DEPs ability seems to be strongly dependent on a range of variables. However, the latest scientific findings indicate that DEPs are likely involved in exacerbations of already existing disease processes, and not in the development of new diseases. The latter hypothesis is based on the enhancement of Th2-dependent responses induced by DEPs [42]. Nonetheless, there are a variety of mechanisms involved in exposure to asthma triggers that do not depend on IgE-mediated responses. These observations have been partially confirmed by epigenetic studies. Epigenetics refers to changes in gene expression that are not caused by alterations in DNA sequences; these changes can be associated with environmental factors such road traffic and smoke pollution [43]. With regard to this issue, recent studies have been focused on mutation hotspots and DNA methylation, involving the so called CpG islands, which are located near the promoter region of many genes. In particular, one of these studies showed significant differences in methylation levels of CpG islands in eosinophils from asthmatic patients compared with non-asthmatic individuals, with the lowest levels of methylation in the subjects with asthma. This study also showed significant associations between IgE and low methylation at 36 loci concentrated in CpG islands [44]. In addition to the above, changes in histone acetyltransferase and deacetylase activity have been observed in cells from patients with lung disorders such as severe asthma and chronic obstructive pulmonary disease (COPD) [45]. Considering that the anti-inflammatory effects of glucocorticoids are dependent on histone deacetylase activity, this finding may be relevant for patients with severe asthma and COPD who are insensitive to glucocorticoid therapy. Indeed, it has been shown that glucocorticoid-insensitive asthmatics have an increased activation of p38 mitogen-activated protein kinase (MAPK), which can reduce the ligand-binding affinity of the glucocorticoid receptor (GR) leading to impaired corticosteroid sensitivity [46]. This can contribute to poor disease control in patients with chronic asthma and COPD. In this respect, it is worth mentioning that also disruption of circadian rhythms may impair asthma control. For example, the degree of asthma control has been shown to strongly correlate with sleep quality. In fact, individuals whose asthma is not well controlled take longer to fall asleep, awake more often, and spend more time awake during the night compared to those with well controlled asthma [47]. Interestingly, many other immunological and allergic diseases, including allergic rhinitis, atopic eczema, chronic urticaria, and rheumatoid arthritis, show a circadian rhythm in the intensity of symptoms and disease severity, which suggests the importance of chronotherapy as a strategy to achieve symptoms control [47].

Of note, not all studies support the hypothesis that air pollution has a greater deleterious effect on asthmatic patients compared to non-asthmatic individuals. However, experimental and clinical studies have demonstrated that asthmatic patients have an increased inflammatory response to air pollutants like PM2.5-10 [48] and especially ozone [49]. Several factors may contribute to the adverse effects of ozone in asthmatic patients by impairing innate immune responses [50].

Epidemiological studies indicate that older people are at higher risk of hospitalisation. In particular, increased exposure to air pollution has been linked to increased mortality, hospital admissions and emergency-room visits in the elderly [51]. These effects are mainly due to exacerbations of chronic diseases or to respiratory tract infections (e.g., pneumonia), and may be modulated by ambient temperature as demonstrated by several studies showing that the elderly are particularly vulnerable to heat waves. In general, it seems that elderly subjects are more vulnerable to PM than to other pollutants, with greater effects on cardiorespiratory mortality and acute hospital admissions [51]. The elderly are at greater risk than other age groups due to their enhanced susceptibility to air pollutants as a result of normal and pathological ageing and related processes. In fact, ageing has been associated with a decline in immune defences and respiratory function, resulting in a higher predisposition to respiratory infections. Moreover, among the chronic diseases that affect the elderly population, COPD and asthma can accelerate pulmonary function decline and increase the risk of mortality [52].

The relationship between short-term exposure to air pollution and cardiorespiratory morbidity and mortality in the elderly is well-documented. A systematic review and meta-analysis conducted by Bell et al. [53] found strong, consistent evidence of a higher risk of PM-associated hospitalisation and death in the elderly. The results of this meta-analysis showed that a 10 μg/m3 increase in PM10 exposure was associated with a higher risk of death of 0.64% (95% confidence interval (CI) 0.50, 0.78) for older populations compared with 0.34% (95% CI 0.25, 0.42) for younger populations. In another systematic review by Bell et al. [54], regarding sensitivity to mortality or hospital admission from short-term ozone exposure, mortality risk was also higher for older persons (1.27%; 95% CI 0.76–1.78) than for younger persons (0.60%; 95% CI 0.40–0.80).

Further evidence of an association between air pollution and mortality in the elderly was provided by another study, which showed that a 3–4% increase in daily deaths for all causes and for cardiovascular disease in the elderly was associated with an increase in fine particulate matter and sulfur dioxide from the 10th to the 90th percentile [55]. The increase in mortality was even higher (6%) for respiratory deaths. Cardiovascular deaths were also associated with levels of carbon monoxide (4% increase in daily deaths). On the contrary, the associations between air pollution and mortality were not statistically significant in children under 5 years. In summary, there was a significant trend of increased risk of death based on age, with effects more evident for individuals over 65 years old. The effect of air pollution was even larger in areas with higher socioeconomic level [55].

With regard to the effects of socioeconomic vulnerability on the risk of mortality in populations exposed to high PM concentrations, an Asian study showed that economic inactivity had the greatest influence on the risk of mortality (Table 1) [56]. People who were economically inactive had a 5.5% higher risk of all-cause mortality and a 5.6% higher risk of cardiorespiratory mortality from a 10 μg/m^3^ increase in PM10–2.5 compared with those who were employed during a day with an extreme dust event. Also, the elderly and non-married people had higher risks of mortality during days with extreme dust events. People aged 65 years and older had a 4.4% higher risk of all-cause mortality and a 5.3% higher risk of cardiorespiratory mortality compared with those who were under the age of 65. People who were not married had a 4.1% higher risk of all-cause mortality and a 4.7% higher risk of cardiorespiratory mortality than those married [56].

**Table 1:** Effect modification of socioeconomic vulnerability for 10 mg/m^3^ increase of PM10-2.5, with 95% confidence interval. Modified from Ho et al. [56]

Socioeconomic vulnerability (individual level)	OR at lag 0 (all cause mortality)	OR at lag 0 (cardiorespiratory mortality)
Age		
> 65	*1.044 [1.029, 1.059]*	1.053 [1.029, 1.078]
< 65	1.047 [0.994, 1.103]	1.045 [0.920, 1.186]
Gender		
Female	1.057 [1.014, 1.102]	1.062 [0.998, 1.131]
Male	1.033 [1.018, 1.049]	1.030 [1.009, 1.052]
Socioeconomic status		
Economically inactive	*1.055 [1.039, 1.070]*	*1.056 [1.030, 1.081]*
Economically active	1.024 [0.992, 1.057]	1.019 [0.974, 1.066]
Marital status		
Non-married	*1.041 [1.018, 1.065]*	*1.047 [1.004, 1.093]*
Married	1.034 [1.013, 1.056]	1.030 [1.003, 1.056]

Italic text indicates results with significant difference between vulnerable and non-vulnerable groups

Importantly, it has been observed that antioxidant metabolic pathways become increasingly compromised with ageing. Older people are also particularly sensitive to the short-term effects of ozone exposure [50]. It is becoming increasingly clear that ageing is strongly associated with senescence of the immune system [57–61]. To this regard, outdoor traffic pollution exposure is the most important predictive factor for asthma in the elderly [62–66]. Indeed, higher levels of outdoor and indoor exposures to DEPs, combined with obesity and atopic state, are associated with inadequate asthma control in the elderly [67]. There is also evidence that heat waves are associated with an increased risk of hospitalisation and higher mortality in the elderly [68]. In particular, two studies have shown that severe cardiovascular and respiratory diseases can be induced by the elevated air pollution associated with heat waves, especially in the elderly [69, 70].

Environmental pollution exposure significantly increases the risk of respiratory diseases, particularly asthma. Both adult and young patients, smokers or non-smokers, are prone to acute asthma attacks as a result of greater exposure to air pollution. In particular, children from low-income backgrounds have been found to be more susceptible to acute asthma attacks due to air pollution. However, larger studies are required to confirm the relevance of these interactions, and to further explore the risk factors for asthma associated with long-term air pollution exposure in patients of different ages. Moreover, when differentiating between the effects of various risk factors, it is important to remember that asthma is not a single disease [71]. In addition, the risk of both allergic and non-allergic asthma seems to increase with increasing BMI as previously reported in children [72]. Finally, associations between early-life environmental exposures and asthma have been found in non atopic subjects [73, 74].

Recently, air pollution has also been associated with increased plasma viscosity and cardiac functions abnormalities, mainly due to cardiac arrhythmias [75–77]. Therefore, elderly patients are more susceptible than younger patients to the adverse effects of climate change due to their higher incidence of cardiovascular disease. Another important issue is the relationship between climate change and microbial infections. It has been suggested that the rapid and intense climate changes related to global warming can create conditions that promote the spread of epidemic diseases. For instance, heavy rainfalls increase the availability of breeding places for pests such as bugs and rats, which contaminate drinking water supplies. Moreover, parasitic and viral diseases transmitted by mosquitoes are very sensitive to climate changes [78–80].

Other mechanisms involved in climate change include air temperature increases in urban areas, a phenomenon called “heat island.” This effect is particularly relevant for people living in these areas, especially those who are affected by cardiovascular and/or respiratory diseases. It also has a significant impact on the most fragile population groups (e.g., elderly and poor people). In fact, these people have less access to air conditioning and tend to stay more outdoors (e.g., streets, squares) where they are more exposed to urban overheating. In addition, exposure to outdoor air pollution combined with exposure to allergenic pollen can trigger severe asthma attacks. A reduction in emergency hospital admissions after the implementation of prevention programs supports the view of a causal link between air pollution and exacerbations of chronic respiratory diseases [80].

### The SIAAIC task force on air pollution and climate change: the decalogue “Allergy Safe Tree”

In April 2014, the Italian Society of Allergology, Asthma and Clinical Immunology (Società Italiana di Allergologia, Asma ed Immnunologia Clinica, SIAAIC) established the Task Force (TF) on “Air Pollution and Climate Change.”

**Fig. 1:**
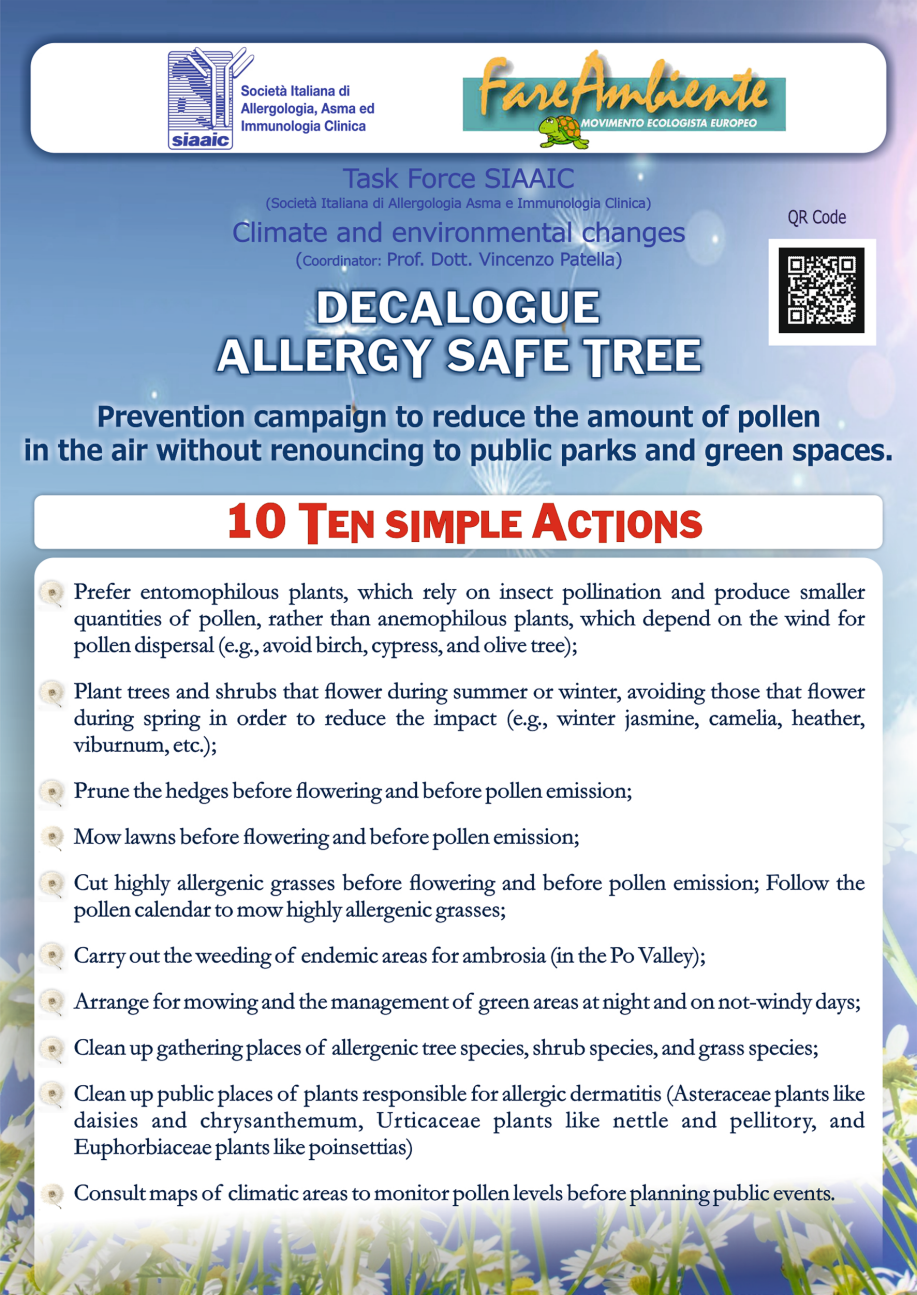
Prevention campaign to reduce the amount of pollen in the air without renouncing to public parks and green spaces

The SIAAIC TF launched a project divided into 3 successive steps. The first step involved the establishment of a multidisciplinary expert group, including high-profile academic, institutional and associative figures working in the field of “Air Pollution and Climate Change” at the legal, environmental and institutional levels. SIAAIC Allergists and Clinical Immunologists were clearly involved in this group. The second step of the project (“exploratory step”) included the collection of national and European data on climate and environmental changes. The third step of the TF’s work, still underway, focused on the preparation of a final document (Report or Guidelines) to raise awareness about “Air Pollution and Climate Change” and their impact on Immunological and Allergic Diseases. The TF also planned the production of a “synthetic” version of the document intended for the public, as well as the preparation of a Decalogue to be sent to local administrators with a set of rules aimed at protecting public health. Starting from this initial proposal, and as a result of its regular activities during these years, the Task Force on Air Pollution and Climate Change of the Italian Society of Allergology, Asthma and Clinical Immunology (SIAAIC) has proposed a National Healthcare Program to help the cities’ public administrations in choosing non allergenic trees in public parks. The “DECALOGUE: ALLERGY SAFE TREE” document suggests how to use non allergenic trees in gardens and public parks [81, 82] (Fig. 1).

As the subtitle indicates, the Decalogue plans to carry out a prevention campaign to reduce the amount of pollen in the air without sacrificing public green areas. To this end, the document adopted by the SIAAIC TF proposes “simple actions” concerning the management and care of plants, targeting several factors affecting allergen concentrations, including the “pollen calendar,” namely the recording of seasonal peaks of airborne pollen concentrations. The SIAAIC TF believes that compliance with the provisions of the Decalogue can help meet the campaign goal, that is, reducing the concentrations of airborne allergenic pollen in the cities. Among the forthcoming activities, the TF is preparing aerobiology courses, as well as the creation of a national SIAAIC aerobiology network for the monitoring of allergies across the country.

Behind the initiatives of the SIAAIC TF on “Air Pollution and Climate Change,” there is the authors’ opinion, which is supported by recent studies, that the prevention of allergies can be implemented by public administrations through a careful choice of non allergenic trees and plants. Likewise, a careful selection of ornamental plants can contribute to reduce pollen levels in the private sector (condos, sports, malls and industrial green areas) [77]. These aspects are of high relevance as parks and gardens are primarily visited by both children and elderly people, who are susceptible to allergic sensitisation and respiratory diseases. Finally, allergen exposure within the parks is particularly dangerous when combined with climate conditions characterized by rising temperatures that can promote pollen production, taking into account the fact that urbanization and high levels of vehicle emissions correlate with a higher frequency of pollen-induced respiratory allergy [4].

## Conclusion

The effects of global warming on human health are supported by many official documents which emphasize the importance of air pollution on the rising incidence of many respiratory diseases. Climate change, by modifying major climatic variables, influences directly and indirectly the formation of air pollutants. Both global warming and solar radiation contribute to the formation of secondary pollutants in the atmosphere, especially tropospheric ozone. All these factors can explain, at least partly, the increasing incidence of respiratory and allergic diseases in different parts of the world. Recent forecasts suggest that climate change will persist for several decades (IPCC 2013). It is likely that we will face the impact of climate change for at least the next 50 years. The effects of climate change are already here and their negative consequences cannot be easily avoided or prevented. In many patients with respiratory diseases like asthma, symptomatic treatment with bronchodilators or inhaled corticosteroids is sufficient to control disease. However, there are patients who do not achieve control despite treatment with the best current therapy. This heterogeneity of asthma and allergic diseases is increasingly appreciated based primarily on recent cluster analyses, molecular phenotyping, biomarkers and differential responses to targeted and non-targeted therapies [83]. This can be explained, at least in part, by different patient exposure to climate change and air pollution. A joint effort among scientists of different disciplines together with public administrations is highly desirable to limit present and future harm to human health as a result of interaction between climate change and air pollution.
